# Supplementary material for: Fast-spiking interneuron detonation drives high-fidelity inhibition in the olfactory bulb
Source: bioRxiv. 2024 May 8:2024.05.07.592874. Preprint. [Version 1] doi: 10.1101/2024.05.07.592874 (PMC11100763; doi:10.1101/2024.05.07.592874)
Supplement: Supplement 1 [file NIHPP2024.05.07.592874v1-supplement-1.pdf]

bioRxiv preprint doi: https://doi.org/10.1101/2024.05.07.592874; this version posted May 8, 2024. The copyright holder for this preprint (which was not certified by peer review) is the author/funder, who has granted bioRxiv a license to display the preprint in perpetuity. It is made available under aCC-BY 4.0 International license.

Supplementary tables

Table S1. EPL-IN intrinsic biophysical properties

	FSI			RSI					
	**mean ± SEM**	**median (Q_1, Q_3)**	**N**	**mean ± SEM**	**median (Q_1, Q_3)**	**N**	**sig.**	**p**	**test value**
Resting properties									
resting potential (mV)	–81.9±0.5	–82.8 (–84.5, –79.7)	95	–78.5±1.6	–81.5 (–84.2, –73.9)	27	n.s.	0.1	r.s. = 1907.5
input resistance (MΩ)	146.4±5.5	137.8 (110.0, 178.8)	92	422.1±30.9	373.3 (288.8, 523.9)	27	***	1.0×10^{-14}	r.s. = 2840
membrane time constant (ms)	6.4±0.2	6.2 (5.1, 7.2)	92	17.2±1.2	17.1 (13.0, 19.9)	27	***	1.5×10^{-13}	r.s. = 2785
membrane capacitance (pF)	46.0±1.2	43.7 (37.1, 54.0)	92	42.8±2.6	43.6 (36.3, 52.2)	27	n.s.	0.4	r.s. = 1494
spontaneous firing rate (Hz)	0.2±0.1	0.02 (0.0, 0.1)	95	0.04±0.03	0.0 (0.0, 0.02)	27	**	3.1×10^{-3}	r.s. = 1206
spontaneous EPSP half-width (ms)	4.2±0.1	4.1 (3.8,4.5)	94	9.2±0.9	7.7 (7.2, 9.0)	22	***	8.3×10^{-13}	r.s. = 2303.5
spontaneous EPSP rate (Hz)	58.7±1.5	59.0 (48.7, 68.0)	95	30.7±1.9	30.4 (23.5, 37.5)	27	***	3.7×10^{-16}	t_{120} = 9.4
spontaneous EPSP median amp. (mV)	1.8±0.1	1.8 (1.4, 2.1)	95	0.5±0.0	0.5 (0.5, 0.7)	27	***	2.7×10^{-14}	r.s. = 426
spontaneous EPSP median rise (ms)	0.6±0.0	0.6 (0.5,0.6)	94	0.9±0.0	0.9 (0.8, 1.1)	23	***	3.6×10^{-13}	r.s. = 2417.5
Step current-evoked spike properties									
spike amp. (mV)	63.6±1.5	63.2 (53.6, 74.0)	92	52.3±2.9	52.3 (42.8, 61.0)	23	**	1.2×10^{-3}	t_{113} = 3.3
spike width (ms)	0.4±0.0	0.4 (0.4, 0.5)	92	0.7±0.0	0.7 (0.6, 0.8)	23	***	2.6×10^{-10}	r.s. = 2210.5
spike threshold (mV)	–50.0±0.8	–50.1 (–55.3, –45.3)	92	–35.3±1.6	–35.7 (–38.8, –30.4)	23	***	4.5×10^{-13}	t_{113} = 8.2
max. spike rising slope (mV/ms)	284.6±10.6	269.5 (212.3, 353.0)	92	179.7±18.5	163.1 (114.5, 228.2)	23	***	1.5×10^{-5}	r.s. = 715
max. spike falling slope (mV/ms)	–187.3±6.1	–182.8 (–230.5, –143.6)	92	–89.8±8.4	–84.4 (–103.9, –63.9)	23	***	6.0×10^{-10}	r.s. = 2220
afterhyperpolarization amp. (mV)	19.9±0.6	20.0 (16.4, 24.1)	92	23.8±1.0	22.4 (19.9, 26.4)	23	**	1.5×10^{-3}	t_{113} = 3.3
afterhyperpolarization 50% decay (ms)	5.9±0.5	4.6 (3.2, 7.2)	92	41.7±11.8	18.2 (9.9, 54.4)	22	***	2.1×10^{-8}	r.s. = 2046
Pulse-evoked spike properties									
afterdepolarization amp. (mV)	1.7±0.1	1.4 (0.6, 2.7)	93	0.4±0.4	0.0 (0.0, 0.0)	22	***	1.7×10^{-8}	r.s. = 492
Firing rate-current (FI) curve properties									
rheobase (pA)	166.1±9.7	150.0 (100.0, 200.0)	93	98.9±12.3	100.0 (35.0, 150.0)	27	***	9.2×10^{-4}	r.s. = 1114
max. gain (Hz/pA)	2.1±0.1	2.0 (1.4, 2.6)	93	0.9±0.2	0.6 (0.4, 1.0)	27	***	4.9×10^{-9}	r.s. = 702
max. instantaneous rate (Hz)	243.6±5.7	238.1 (204.1, 277.8)	93	147.9±12.1	149.3 (99.5, 180.2)	27	***	3.7×10^{-12}	t_{118} = 7.7
max. interspike interval (ISI) C.V.	2.2±0.1	2.3 (1.4, 3.1)	92	0.3±0.0	0.3 (0.2, 0.4)	27	***	9.6×10^{-14}	r.s. = 446
relative adaptation (ISI_{first}/ISI_{last}; %)	94.5±1.9	92.4 (86.8, 101.4)	88	58.4±5.2	49.9 (41.0, 73.7)	27	***	2.3×10^{-8}	r.s. = 719
absolute adaptation ($ISI_{last}–ISI_{first}$; ms)	0.7±0.2	0.7 (–0.1, 1.2)	88	26.0±4.4	22.4 (9.6, 44.2)	27	***	1.4×10^{-9}	r.s. = 2484

bioRxiv preprint doi: https://doi.org/10.1101/2024.05.07.592874; this version posted May 8, 2024. The copyright holder for this preprint (which was not certified by peer review) is the author/funder, who has granted bioRxiv a license to display the preprint in perpetuity. It is made available under aCC-BY 4.0 International license.

Table S2. EPL-IN anatomical and morphometric properties

	FSI mean ± SEM	FSI median (Q_1, Q_3)	FSI N	RSI mean ± SEM	RSI median (Q_1, Q_3)	RSI N	sig.	p	test value
relative EPL depth (0=MCL, 1=GL)	0.59±0.02	0.62 (0.49, 0.71)	104	0.65±0.02	0.63 (0.55, 0.76)	28	n.s.	0.2	r.s. = 6659
soma area (µm²)	94.2±5.2	97.0 (77.9, 104.8)	16	78.6±5.9	73.8 (61.6, 96.4)	9	n.s.	0.07	t_{23} = 1.9
soma max. diameter (µm)	13.2±0.4	13.2 (12.4, 13.9)	16	13.3±0.8	12.6 (11.4, 15.0)	9	n.s.	0.9	t_{23} = 0.1
soma max. diameter/ min. diameter	1.37±0.06	1.32 (1.16, 1.53)	16	1.48±0.11	1.38 (1.28, 1.50)	9	n.s.	0.3	r.s. = 189
dendritic fractal dimension	1.11±0.01	1.12 (1.08, 1.14)	16	1.08±0.01	1.07 (1.05, 1.09)	9	*	0.01	t_{23} = 2.8
total dendritic length (mm)	1.44±0.07	1.48 (1.21, 1.68)	16	0.84±0.14	0.71 (0.53, 1.14)	9	***	3.6×10^{-4}	t_{23} = 4.2
# primary dendritic branches	4.3±0.5	4.0 (3.0, 5.5)	16	3.9±0.6	4.0 (3.0, 4.0)	9	n.s.	0.7	r.s. = 214.5
# terminal dendritic branches	50.8±4.0	49.5 (38.5, 63.5)	16	15.7±3.8	11.0 (9.8, 21.5)	9	***	2.1×10^{-4}	r.s. = 274
# total dendritic branches	96.4±7.7	95.5 (72.5, 115.5)	16	27.0±7.3	18.0 (15.8, 35.8)	9	***	2.0×10^{-4}	r.s. = 274
dendritic spine density (#/100 µm)	3.2±0.2	3.2 (2.7, 3.8)	16	11.6±2.2	13.7 (4.4, 16.0)	9	***	3.1×10^{-5}	t_{23} = 5.2

Table S3. Template parameters for postsynaptic event detection

Event	baseline (ms)	duration (ms)	amplitude (a.u.)	rise (ms)	decay (ms)	min. separation (ms)	threshold (×S.D.)
MTC IPSC	0.5	5.0	–1	0.8	4.0	0.5	2.5
MTC IPSP	1.0	8.0	–1	1.0	6.0	0.5	3.0
EPL-IN EPSP	0.5	5.0	1	0.7	4.0	0.5	2.5

bioRxiv preprint doi: https://doi.org/10.1101/2024.05.07.592874; this version posted May 8, 2024. The copyright holder for this preprint (which was not certified by peer review) is the author/funder, who has granted bioRxiv a license to display the preprint in perpetuity. It is made available under aCC-BY 4.0 International license.

Supplementary figures

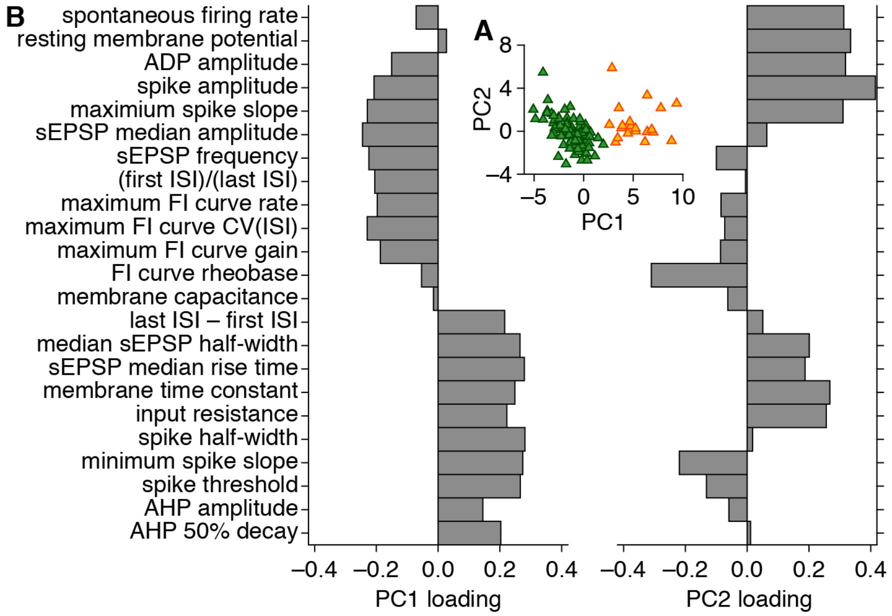

Figure S1. Principal component analysis reinforces subdivision of EPL-INs into FSIs and RSIs
A: Projection of EPL-INs onto the first two principal components (PC1 and PC2) defined by principal component analysis of z-scored intrinsic biophysical properties, revealing two major clusters matching FSI (green) and RSI (orange) subtypes. **B:** Decomposition of PC1 and PC2 loading by each intrinsic biophysical property.

bioRxiv preprint doi: https://doi.org/10.1101/2024.05.07.592874; this version posted May 8, 2024. The copyright holder for this preprint (which was not certified by peer review) is the author/funder, who has granted bioRxiv a license to display the preprint in perpetuity. It is made available under aCC-BY 4.0 International license.

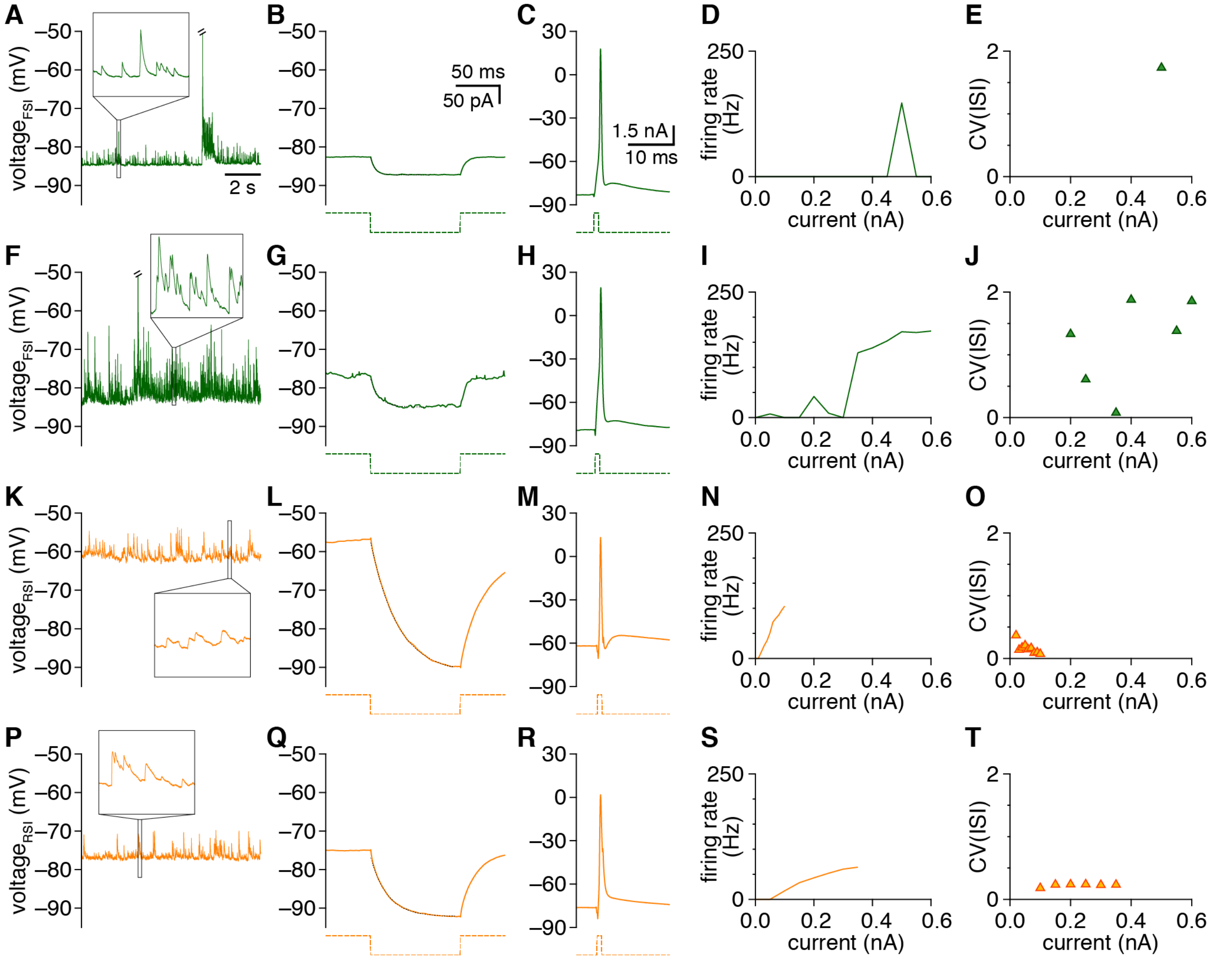

Figure S2. EPL-IN subtypes exhibit stark differences in most intrinsic biophysical properties

A-E: Diverse responses used to calculate intrinsic biophysical properties for the example FSI from Figure 1A, including: spontaneous activity at resting membrane potential (**A**), mean response to negative step current injection, with single-exponential fit (dashed black line) (**B**), mean spike waveform evoked by 1-ms suprathreshold current injection (**C**), firing rate-current relationship (**D**), and interspike interval (ISI) coefficient of variation evoked by positive step current injection (**E**). Spontaneous spike in **A** truncated to better visualize synaptic activity. Inset in **A**: magnification of spontaneous synaptic events. **F-T:** Same as **A-E** for the example FSI and RSIs from Figure 1C,E,G. Insets in **A,F,K,P** are identically scaled.

bioRxiv preprint doi: https://doi.org/10.1101/2024.05.07.592874; this version posted May 8, 2024. The copyright holder for this preprint (which was not certified by peer review) is the author/funder, who has granted bioRxiv a license to display the preprint in perpetuity. It is made available under aCC-BY 4.0 International license.

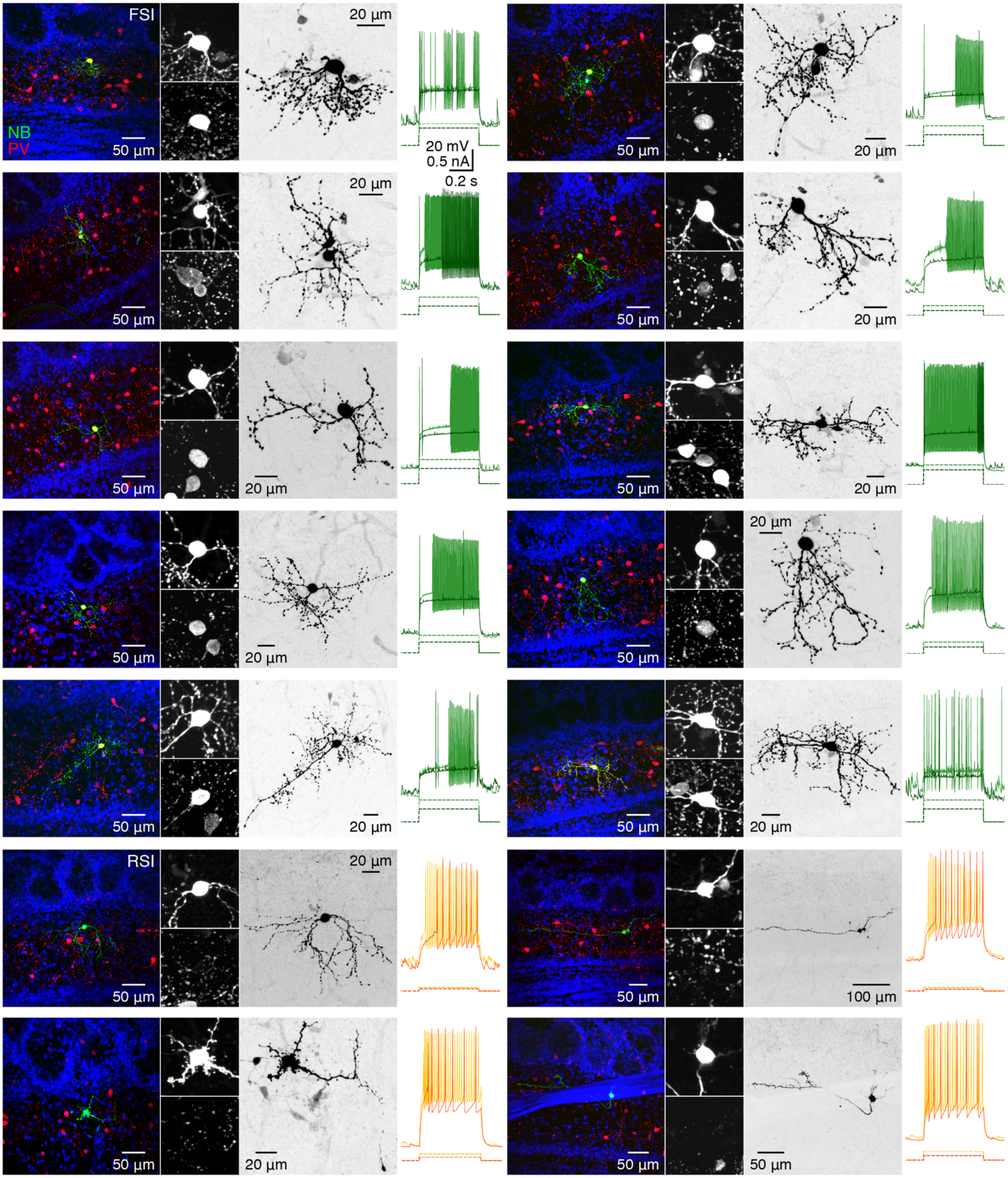

Figure S3. PV expression distinguishes FSIs from RSIs
Intracellular NB and post hoc PV staining with 50-µm magnified inset of somata (left), inverted NB (middle), and step current-evoked spiking (right) of a panel of EPL-INs. Spiking responses are color-coded to reflect FSI vs. RSI physiology, as in Figure 1.

bioRxiv preprint doi: https://doi.org/10.1101/2024.05.07.592874; this version posted May 8, 2024. The copyright holder for this preprint (which was not certified by peer review) is the author/funder, who has granted bioRxiv a license to display the preprint in perpetuity. It is made available under aCC-BY 4.0 International license.

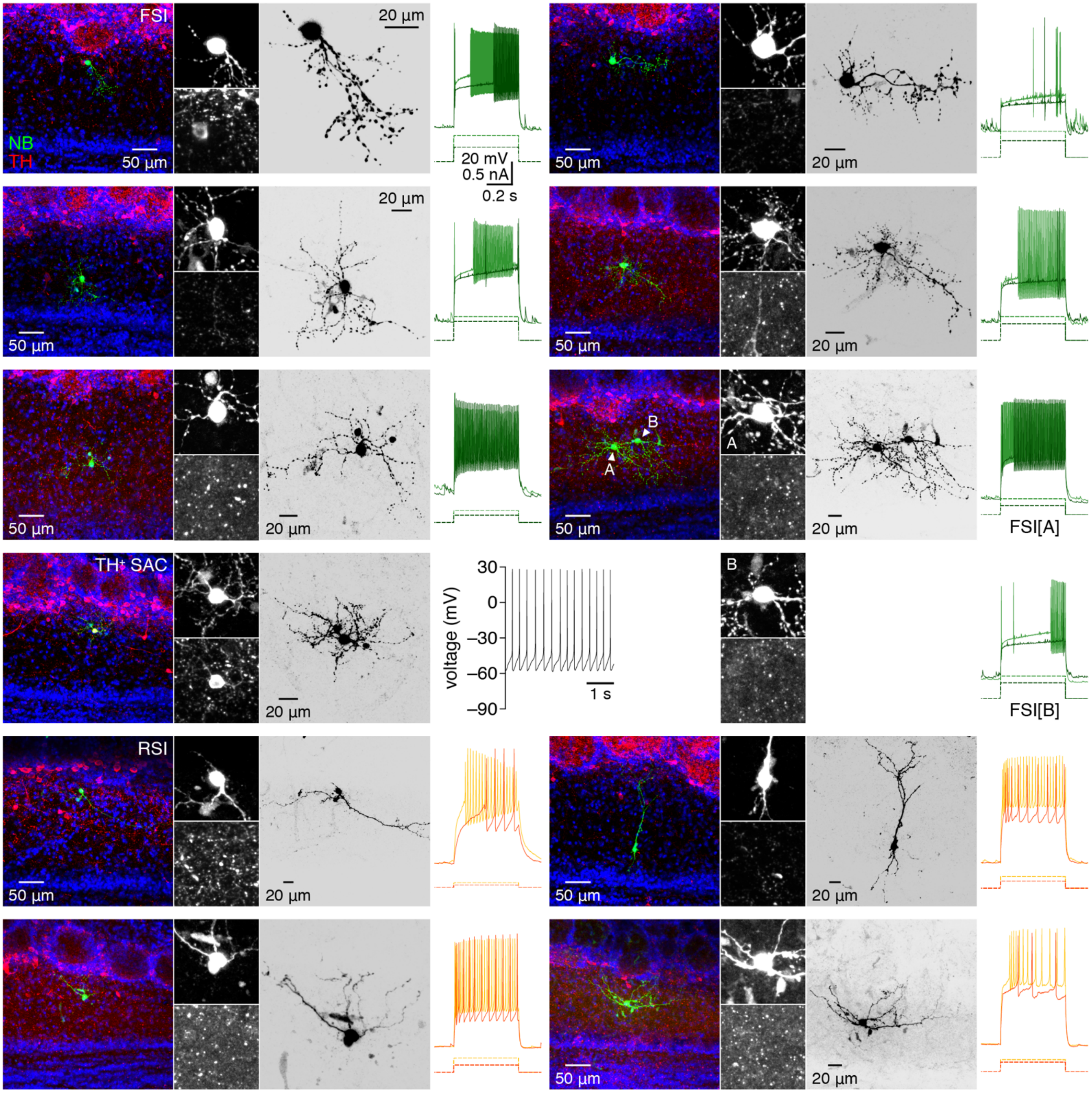

Figure S4. Neither FSIs nor RSIs are dopaminergic

Intracellular NB and post hoc TH staining with 50-µm magnified inset of somata (left), inverted NB (middle), and step current-evoked spiking (right) of a panel of EPL-INs. Spiking responses are color-coded to reflect FSI vs. RSI physiology, as in Figure 1. An example TH+ SAC exhibiting tonic spontaneous firing is additionally included as positive control for TH staining.

bioRxiv preprint doi: https://doi.org/10.1101/2024.05.07.592874; this version posted May 8, 2024. The copyright holder for this preprint (which was not certified by peer review) is the author/funder, who has granted bioRxiv a license to display the preprint in perpetuity. It is made available under aCC-BY 4.0 International license.

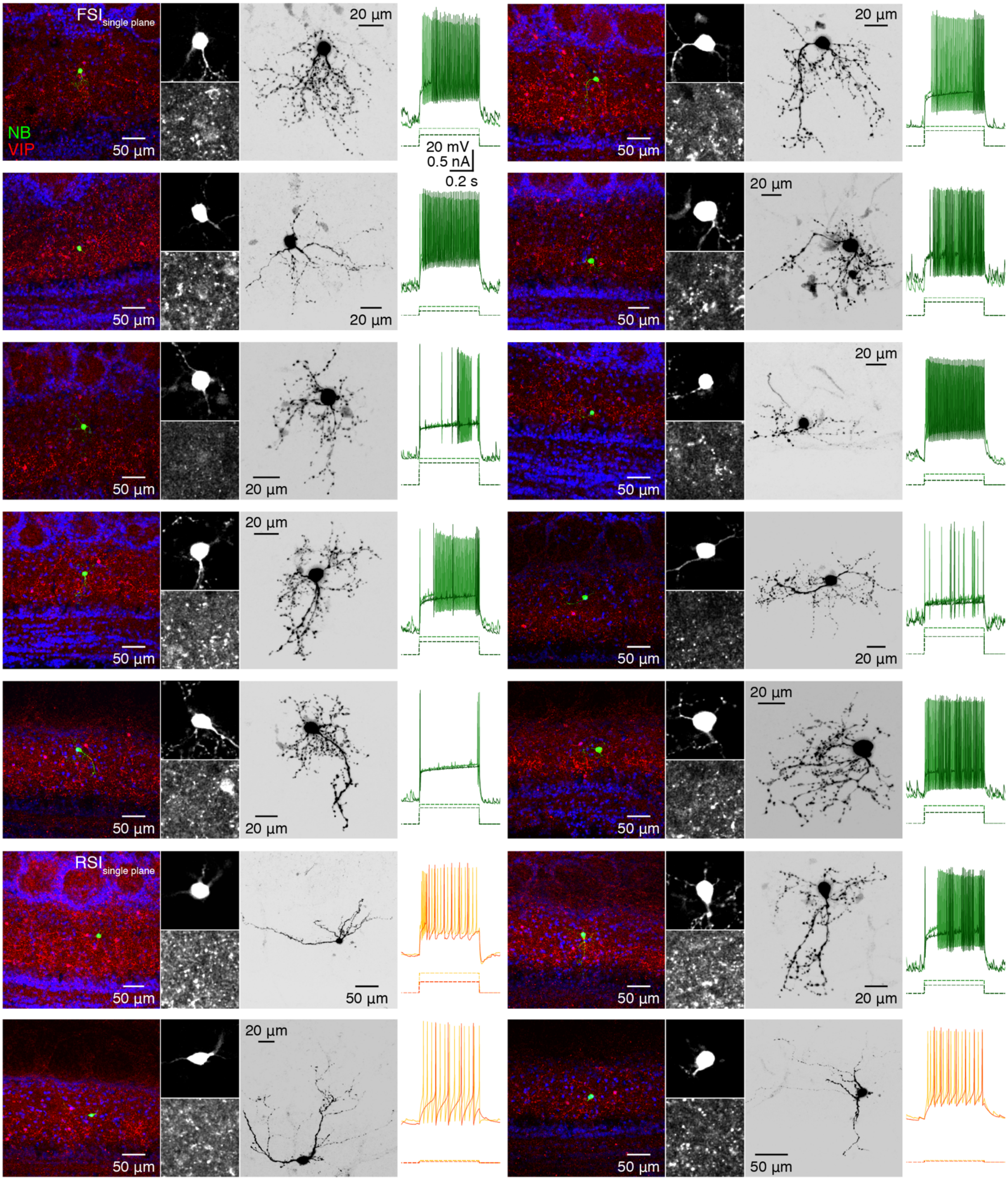

Figure S5. VIP expression poorly distinguishes FSIs and RSIs

Intracellular NB and post hoc VIP staining with 50-µm magnified inset of somata (left; single optical confocal planes), inverted NB (middle; maximum-intensity confocal projection), and step current-evoked spiking (right) of a panel of EPL-INs. Spiking responses are color-coded to reflect FSI vs. RSI physiology, as in Figure 1.

bioRxiv preprint doi: https://doi.org/10.1101/2024.05.07.592874; this version posted May 8, 2024. The copyright holder for this preprint (which was not certified by peer review) is the author/funder, who has granted bioRxiv a license to display the preprint in perpetuity. It is made available under aCC-BY 4.0 International license.

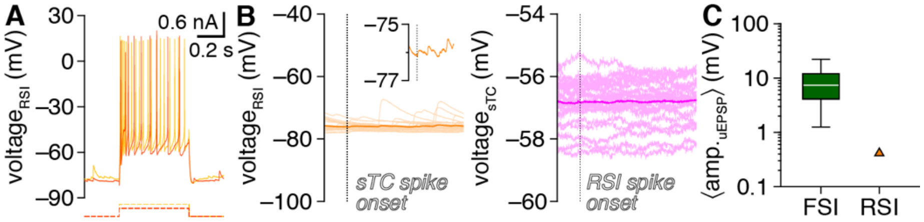

Figure S6. Unitary MTC-to-RSI excitation in a solitary example was distinctly weaker than FSI excitation
A,B: Step current-evoked spiking response (**A**) and unitary synaptic interactions (**B**) for the solitary MTC–RSI pair exhibiting significant unitary MTC-to-RSI excitation (morphology not recovered). Postsynaptic RSI voltage shown on same scale as Figure 1O,P for comparison to postsynaptic FSI responses. Inset: magnification of mean postsynaptic RSI voltage. **C:** The MTC-to-RSI uEPSP amplitude was markedly weaker than FSI uEPSPs (n=69).

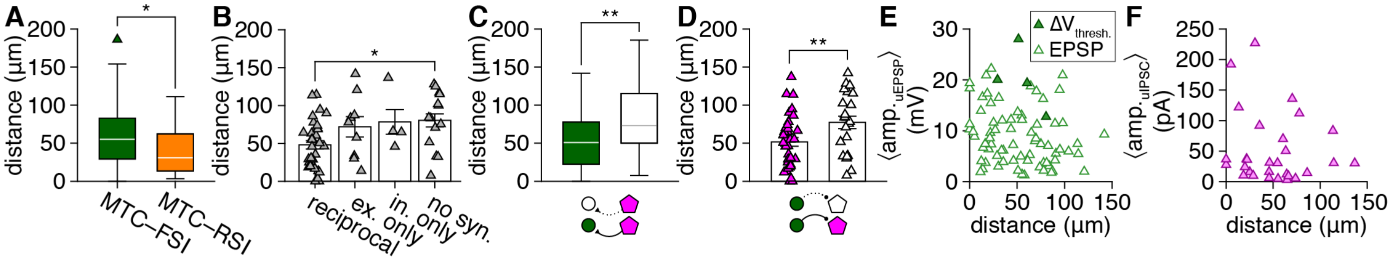

Figure S7. Connected MTC–FSI pairs exhibit shorter intersomatic distances than unconnected pairs
A: MTC–RSI pairs (n=19) exhibited modestly shorter intersomatic distances than MTC–FSI pairs (n=97) (*p=0.02, Wilcoxon rank-sum test). **B:** Among MTC–FSI pairs, reciprocally-connected pairs exhibited shorter intersomatic distances than unconnected pairs (p=0.02, F3,55=3.4, one-way ANOVA; reciprocal vs. excitation only: p=0.3, reciprocal vs. inhibition only: p=0.4, reciprocal vs. unconnected: p=0.03, excitation only vs. inhibition only: p=1.0, excitation only vs. unconnected: p=0.9, inhibition only vs. unconnected: p=1.0, post-hoc Tukey-Kramer test). **C:** MTC–FSI pairs with significant unitary MTC-to-FSI excitation exhibited shorter intersomatic distances than pairs with no excitatory connectivity (**p=2.5×10⁻³, Wilcoxon rank-sum test). **D:** MTC–FSI pairs with significant unitary FSI-to-MTC inhibition exhibited shorter intersomatic distances than pairs with no inhibitory connectivity (**p=9.7×10⁻³, t_{57}=2.7, two-sample t-test). Analysis restricted to pairs with voltage-clamped MTCs (and therefore sensitive detection of unitary inhibition). **E,F:** Neither MTC-to-FSI uEPSP amplitudes (**E**) nor FSI-to-MTC uIPSC amplitudes (**F**) correlated with intersomatic distance (uEPSP: n=77 connections; p=0.1, t_{75}=1.7, linear regression, slope not significantly different from 0; uIPSC: n=29 connections; p=0.4, t_{27}=0.8, linear regression, slope not significantly different from 0). Pairs lacking connectivity (i.e., uEPSP or uIPSC amplitude of zero) not included in analysis.

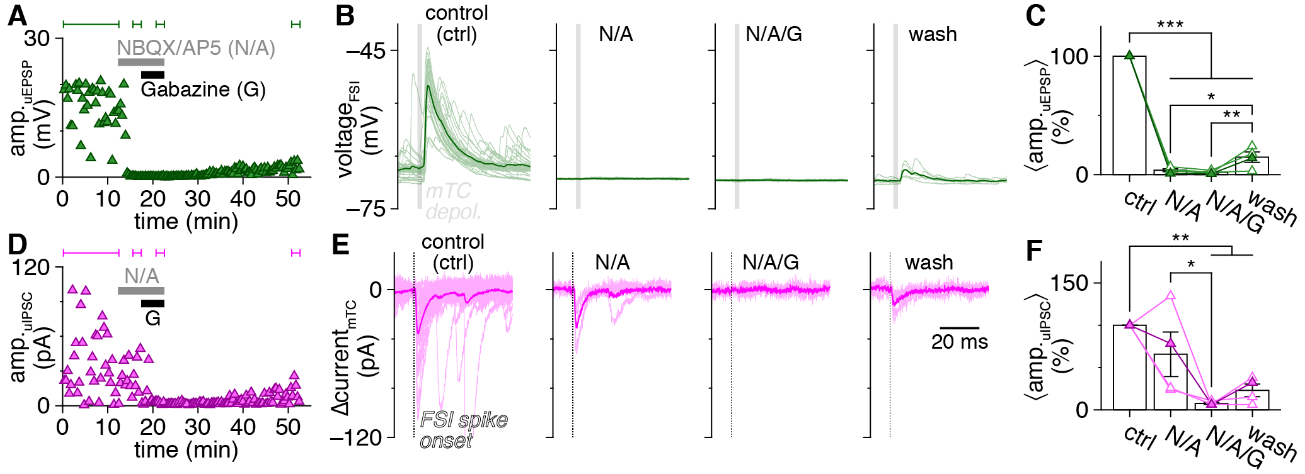

Figure S8. Unitary MTC–FSI synaptic pharmacology
A: Recording from an example MTC–FSI pair (morphology not recovered) showing MTC-to-FSI uEPSP amplitudes before and after combined bath application of glutamatergic antagonists NBQX (10 µM) and AP5 (50 µM) and subsequent application of $GABA_AR$ antagonist Gabazine (10 µM). **B:** Postsynaptic FSI voltages from the pair in **A**. Traces in each subplot correspond to the bracketed trials in **A**. **C:** Unitary MTC-to-FSI excitation was blocked by combined application of NBQX and AP5 and partially recovered upon wash-out in 4 MTC–FSI pairs

bioRxiv preprint doi: https://doi.org/10.1101/2024.05.07.592874; this version posted May 8, 2024. The copyright holder for this preprint (which was not certified by peer review) is the author/funder, who has granted bioRxiv a license to display the preprint in perpetuity. It is made available under aCC-BY 4.0 International license.

($p=2.2\times10^{-12}$, $F_{3,12}=414.9$, one-way ANOVA; ctrl vs. N/A: ***$p=5.5\times10^{-9}$, ctrl vs. N/A/G: ***$p=5.5\times10^{-9}$, ctrl vs. wash: ***$p=5.5\times10^{-9}$, N/A vs. N/A/G: $p=0.9$, N/A vs. wash: *$p=0.03$, N/A/G vs. wash: **$p=0.01$, post-hoc Tukey-Kramer test). **D,E:** Same as **A,B** for FSI-to-MTC uIPSCs recorded in the same example pair. **F:** Unitary FSI-to-MTC inhibition was blocked by application of Gabazine and partially recovered upon wash-out in the same 4 MTC–FSI pairs as **C** ($p=1.7\times10^{-3}$, $F_{3,12}=9.5$, one-way ANOVA; ctrl vs. N/A: $p=0.33$, ctrl vs. N/A/G: **$p=2.1\times10^{-3}$, ctrl vs. wash: **$p=8.3\times10^{-3}$, N/A vs. N/A/G: *$p=0.046$, N/A vs. wash: $p=0.17$, N/A/G vs. wash: $p=0.85$, post-hoc Tukey-Kramer test). Filled symbols in **C,F** correspond to the example pair shown.

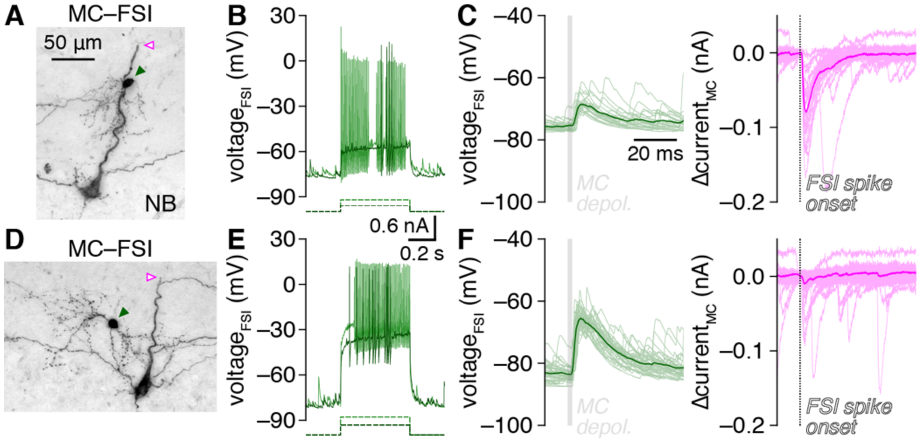

Figure S9. MTC–FSI connectivity is restricted to infraglomerular layers

A: Example MTC–FSI pair with MTC apical dendrite truncated prior to entering glomerular layer (open arrowhead). **B,C:** FSI fast-spiking response to step current injection (**B**) and unitary synaptic connectivity with MTC (**C**). **D-F:** Same as **A-C** for a second example MTC–FSI pair.

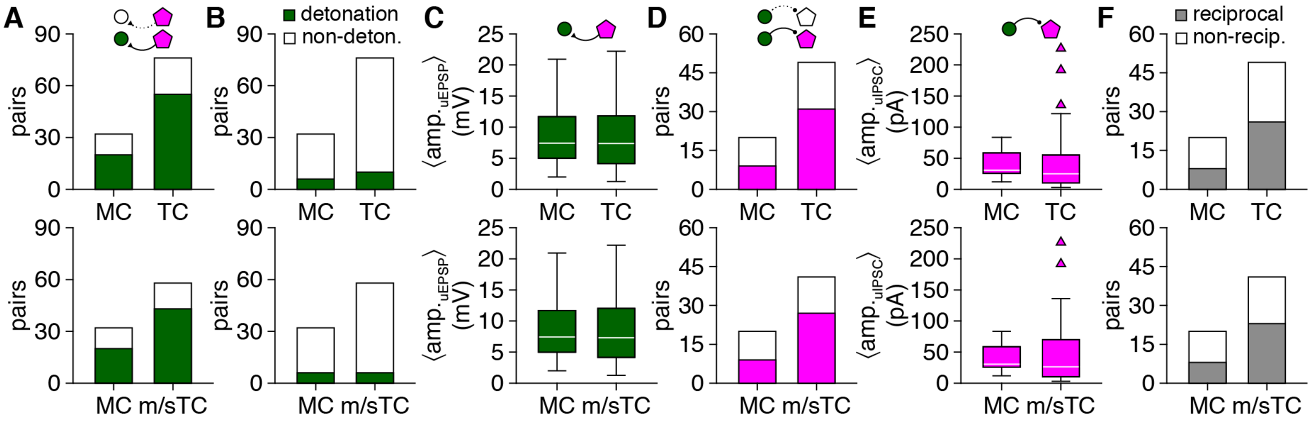

Figure S10. MCs and TCs exhibit similar unitary connectivity with FSIs

A: Detection of unitary FSI excitation did not significantly differ between MC–FSI and TC–FSI pairs (upper; $p=0.3$, $\chi^2_{[1]}=1.0$, χ^2 test) even when considering only middle and superficial TCs (m/sTCs) to exclude potential misclassification of deep TCs (lower; $p=0.2$, $\chi^2_{[1]}=1.3$, χ^2 test). **B:** The proportion of FSIs responding to unitary MTC release with detonation did not significantly differ between MC–FSI and TC–FSI pairs (upper; $p=0.5$, $\chi^2_{[1]}=0.6$, χ^2 test) or between MC–FSI and m/sTC–FSI pairs (lower; $p=0.3$, $\chi^2_{[1]}=1.3$, χ^2 test). **C:** FSI uEPSP amplitudes did not significantly differ between MC-FSI and TC-FSI pairs (upper; $p=1.0$, Wilcoxon rank-sum test) or between MC–FSI and m/sTC–FSI pairs (lower, $p=1.0$, Wilcoxon rank-sum test). **D:** Detection of unitary MTC inhibition did not significantly differ between MC–FSI and TC–FSI pairs (upper; $p=0.2$, $\chi^2_{[1]}=1.9$, χ^2 test) or between MC–FSI and m/sTC–FSI pairs (lower; $p=0.1$, $\chi^2_{[1]}=2.4$, χ^2 test); only voltage-clamped MTCs were considered for peak detection sensitivity. **E:** MTC uIPSC amplitudes did not significantly differ between MC–FSI and TC–FSI pairs (upper; $p=0.6$, Wilcoxon rank-sum test) or between MC–FSI and m/sTC–FSI pairs (lower; $p=0.6$, Wilcoxon rank-sum test). **F:** The proportion of MTC–FSI pairs exhibiting reciprocal unitary connectivity did not differ between MC–FSI and TC–FSI pairs (upper; $p=0.3$, $\chi^2_{[1]}=1.0$, χ^2 test) or between MC–FSI and m/sTC–FSI pairs (lower; $p=0.2$, $\chi^2_{[1]}=1.4$, χ^2 test).

bioRxiv preprint doi: https://doi.org/10.1101/2024.05.07.592874; this version posted May 8, 2024. The copyright holder for this preprint (which was not certified by peer review) is the author/funder, who has granted bioRxiv a license to display the preprint in perpetuity. It is made available under aCC-BY 4.0 International license.

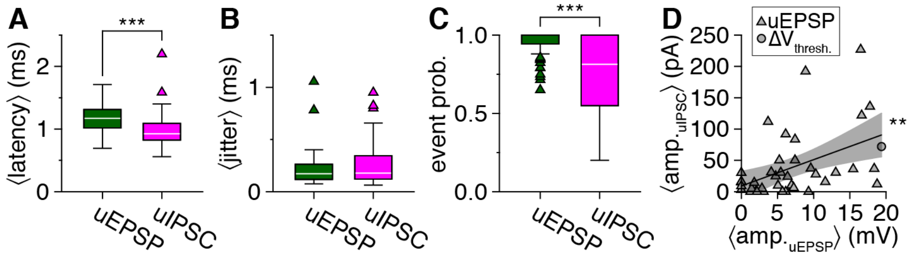

Figure S11. Comparison of unitary FSI-to-MTC and MTC-to-FSI synaptic transmission properties
A: FSI-to-MTC uIPSC latency (n=44 connections) was significantly shorter than MTC-to-FSI uEPSP latency (n=31 connections) (***p=8.0×10^{-5}, Wilcoxon rank-sum test). **B:** FSI-to-MTC uIPSC jitter (n=44 connections) and MTC-to-FSI uEPSP jitter (n=31 connections) were equivalent (p=0.5, Wilcoxon rank-sum test). **C:** Trial-to-trial FSI-to-MTC uIPSC event probability (n=44 connections) was significantly lower than trial-to-trial MTC-to-FSI uEPSP event probability (n=79 connections) (***p=5.4×10^{-8}, Wilcoxon rank-sum test). Unitary FSI-to-MTC IPSP latency, jitter, and probability not included in comparisons due to limited unitary IPSP detection sensitivity (Figure 1U). **D:** Across all MTC–FSI pairs with at least one direction of unitary connectivity, FSI-to-MTC uIPSC amplitude positively correlated with MTC-to-FSI uEPSP amplitude (n=41 pairs; **p=1.9×10^{-3}, t_{39}=3.3, R^2=0.22, linear regression, slope significantly different from 0). For pairs exhibiting exclusive FSI detonation, uEPSP amplitudes were estimated as the difference between resting membrane potential and spike threshold ($\Delta V_{thresh.}$), as in Figure 3G. Shading denotes 95% confidence interval.

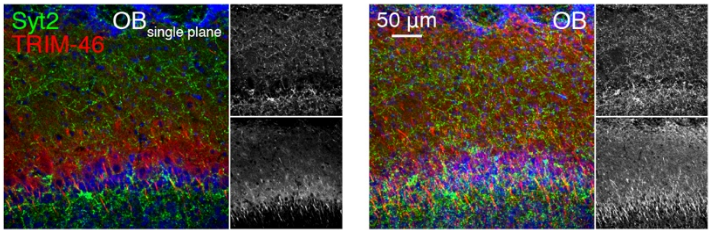

Figure S12. Syt2 clusters do not selectively target MTC axon initial segments
Single confocal optical plane (left) and maximum-intensity projection (right; ~50 µm depth) of Syt2 and axon initial segment component TRIM-46 in the OB, revealing an absence of clear Chandelier-like innervation of MTCs.

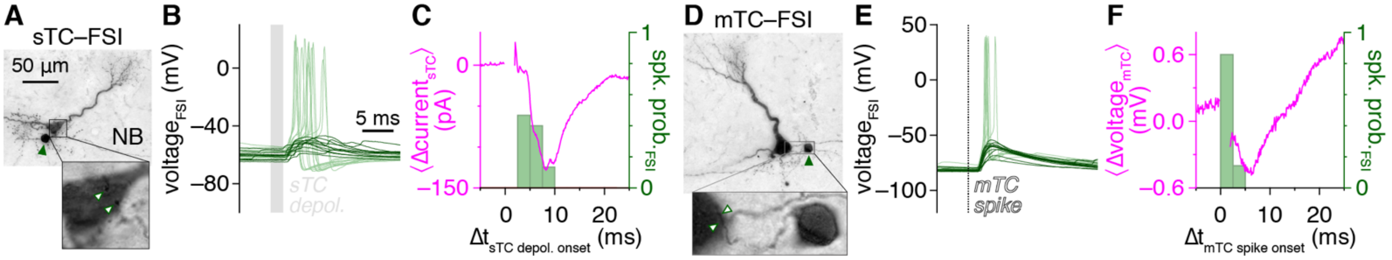

Figure S13. Comparison of spontaneously alternating FSI detonation vs. uEPSP trials reveals high-fidelity recurrent MTC inhibition
A,B: Example MTC–FSI pair (**A**) in which unitary MTC release triggers FSI detonation on some trials (light green) and uEPSPs on other trials (dark green) (**B**). **C:** Subtraction of mean MTC currents across FSI detonation vs. uEPSP trials from **B** isolates IPSC waveforms time-locked to FSI detonation. **D-F:** Same as **A-C** for an example MTC-FSI pair recorded in current-clamp, revealing isolation of an IPSP waveform time-locked to FSI detonation.
